# Supplementary material for: Impact of Different Levels of Supervision on the Recovery of Severely Malnourished Children Treated by Community Health Workers in Mali
Source: Nutrients. 2021 Jan 26;13(2):367. doi: 10.3390/nu13020367 (PMC7911749; doi:10.3390/nu13020367)
Supplement: Supplementary file 1 [file nutrients-13-00367-s001.pdf]

Impact of different levels of supervision on the recovery of severely malnourished children treated by community health workers in Mali –

Supplementary A.

Table A-1 – Checklist for the supervision of iCCM activities

**CLINICAL EXAMINATION OF THE SICK CHILD** (13 items)	**Pass**	**Fail**
1. Handwashing with soap		
2. Weighing of the child		
3. Taking the child's temperature		
4. Use of the MUAC tape		
5. Looking for danger signs		
6. Breathing movement count		
7. Correct application of diagnostic protocols		
8. Correct classification of the child's health status		
9. Administration of the drug 1st dose in front of the mother		
10. Explanations to the mother on how to administer the drugs at home		
11. Checking maternal understanding		
12. Advice on preventive actions on malaria, ARI, diarrhea, MAM & SAM		
13. Fixing the appointment for follow-up		
**NEWBORN MONITORING** (12 items)	**Pass**	**Fail**
1. Handwashing with soap		
2. Look for danger signs in mother and baby		
3. Application of chlorhexidine gel for umbilical cord care		
4. Advice on exclusive breastfeeding of newborn		
5. Advice on supplementary feeding practices		
6. Advice on the use of long-lasting insecticide-treated mosquito nets		
7. Advice on the breastfeeding mother feeding		
8. Family planning counseling		
9. Advice on post-natal consultations		
10. Advice on food, personal and environmental hygiene		

11. Advice on vaccination advice		
12. Advice on how to maintain the temperature of the newborn baby		
**FAMILY PLANNING** (4 items)	**Pass**	**Fail**
1. Handwashing with soap		
2. Compliance with GATHER method steps*		
3. Using the eligibility checklist		
4. Respecting confidentiality		
**INFANT AND YOUNG CHILD FEEDING PRACTICES** (3 items)	**Pass**	**Fail**
1. Compliance with IYCF steps (evaluate, analyze and act)		
2. Respecting the support group facilitation steps		
3. Follow the nutrition demonstration steps		
**SANITATION AND HYGIENE** (7 items)	**Pass**	**Fail**
1. Mastery of the handwashing technique		
2. Good knowledge of the critical handwashing moments		
3. Water treatment by aquatabs		
4. Water treatment with bleach		
5. Water treatment by a filter		
6. Water treatment by decantation		
7. Mastery of the well treatment technique		

*ARI: acute Respiratory Infection; IYCF_ Infant and young child feeding; MAM: moderate acute malnutrition; MUAC: middle upper arm circumference SAM: severe acute malnutrition. *GATHER: Greet, Ask, Tell, Help, Explain and Return; In French is called 'méthode BERCER' (bienveillance, ecoute, rigueur, confiance, engagement et réactivaté).*

Table A-2 – Checklist for the nutrition-specific supervision

**EQUIPMENT AVAILABILITY** (2 items)	**Pass**	**Fail**
1. Anthropometric and nutritional equipment present and in good condition (scales, MUAC tape, ORS kit, etc.)		
2. Medical examination equipment present and in good condition (thermometer, chronometer, RDT, etc)		
**OTHER MATERIAL AVAILABILITY** (5 items)	**Pass**	**Fail**
1. Register sheets		
2. Patient cards (follow-up), growth charts, reference sheets, etc.		
3. Protocols (PECIMA, IYCF, supplementation, etc.)		
4. Educational material and counseling cards		
5. Communication material available and functional (mobile phone)		
**PHARMACY STOCK** (4 items)	**Pass**	**Fail**
1. Adequate, clean and ventilated stock/pharmacy conditions (shelf storage, labelling, use of pallets, rodent free, etc.)		
2. Stock cards for nutritional (RUTF, RUSF, CSB+, Oil), and routine treatment drugs inputs (albendazole, Vit A, Iron, Folic Acid) and iodine testers present and correctly filled in.		
3. Sufficient quantity of nutritional and routine drugs inputs and iodine testers in stock (1 month operation + 2 weeks buffer stock) and no stock-out registered during the last month		
4. Physical inventory carried last monthly		
**SAFE DRINKING WATER** (1 item)	**Pass**	**Fail**
1. Drinking water station available and functional		
**IDENTIFICATION OF DANGER SIGNS** (20 items)	**Pass**	**Fail**
1. Checking if the child is younger than 2 months of age		
2. Visible and severe weight loss in children 2 to 6 months		
3. Is the child unable to drink or suckle?		
4. Does the child vomit everything he/she consumes?		

5. Has the child had a seizure or is convulsing now?		
6. Is the child unconscious or unresponsive to external stimuli?		
7. Oedema assessment of the lower limbs		
8. Severe palmar pallor (anemia)		
9. Difficult breathing with chest tightness or wheezing		
10. Spontaneous bleeding		
11. Urine scarce and like 'coca-cola' color		
12. Unable to sit or stand upright		
13. Child gets sicker despite home care		
14. All illnesses lasting 14 days or more		
15. Signs of dehydration (sunken eyes, thirsty, skinfolds slowly disappearing, restless or irritable child)		
16. MUAC measurement on the red zone		
17. MUAC on the red zone and appetite test failure		
18. Blood in the stool		
19. Stools are too liquid (like water)		
20. The child 2 to 6 months does not gain weight		
**SYSTEMATICALLY SCREENING** (1 items)	**Pass**	**Fail**
1. Passive screening carried out systematically (verified in the logbook of consultations for the last month)		
**ADMISSION & DISCHARGE CRITERIA APPLICATION** (2 items)	**Pass**	**Fail**
1. Admission criteria known, correctly applied and noted on the patient card		
2. Discharge criteria known, correctly applied and noted on the patient card		
**APPETITE TEST PERFORMANCE** (2 items)	**Pass**	**Fail**
1. Correct quantity of RUTF given for passing the test		
2. Monitoring the mother during the test and result noted in the card		
**NUTRITIONAL TREATMENT** (1 items)	**Pass**	**Fail**
1. Correct number of RUTF sachets given and noted on the card		

**SYSTEMATIC MEDICAL TREATMENT** (1 items)	**Pass**	**Fail**
1. Systematic medical protocol correctly applied and noted on the card (correct product, dose and frequency)		
**INFANT AND YOUNG CHILD FEEDING PROMOTION** (3 items)	**Pass**	**Fail**
1. Promotes and advises on IYCF to the caregiver and refers him/her to the support groups during the curative consultation of the under 2 years child		
2. Counselling sessions are carried out for all pregnant women during the medical consultation and follow-up for uncomplicated malaria by respecting the three steps of the IYCF assessment		
3. Nutritional demonstration sessions are carried out in accordance with the nutritional demonstration steps		

CSB+: Corn Soy Blend +; IYCF: infant and young child feeding; MUAC: middle upper arm circumference; ORS: oral rehydration solution; PECIMA: integrated management of acute malnutrition (in French: prise en Charge Integrée de la malnutrition aiguë); RDT: rapid diagnostic tests for malaria; RUSF: ready-to-use supplementary Food; RUTF: ready-to-use therapeutic food.

Supplementary B

Table B-1 - Average score from a maximum of 10 obtained by the community health workers in Kita and Kayes districts for the curative and preventive tasks assessed on the iCCM supervisions from January to October 2018.

	**KITA** (High supervision) Mean ± SD	**KAYES** (Light supervision) Mean ± SD	p value
**Clinical examination of the sick child**	**9.33 ± 1.00**	**8.81 ± 1.37**	**<0.001**
Handwashing with soap	*8.56 ± 3.42*	*7.96 ± 3.78*	*0.057*
Evaluation of weight	*9.57 ± 1.87*	*9.01 ± 2.74*	*0.011*
Measure temperature	*9.87 ± 0.98*	*9.01 ± 2.65*	*<0.001*
Use of MUAC	*9.59 ± 1.70*	*9.10 ± 2.40*	*0.009*
Danger signs assessment	*9.85 ± 1.08*	*9.48 ± 1.83*	*0.007*
Breath movements assessment	*9.64 ± 1.68*	*9.07 ± 2.63*	*0.006*
Correct use of RDT	*9.56 ± 1.83*	*8.91 ± 2.66*	*0.002*
Correct triage	*9.58 ± 1.68*	*9.27 ± 1.91*	*0.048*
1st dose given in front of the mother	*9.03 ± 2.79*	*8.45 ± 3.17*	*0.030*
Explain how to give drugs at home	*9.24 ± 2.20*	*8.38 ± 3.10*	*<0.001*
Verify understanding	*8.50 ± 3.26*	*8.03 ± 3.40*	*0.103*
Prevention tips	*7.98 ± 3.50*	*8.16 ± 3.31*	*0.535^{NS}*
Fix a date to follow up	*9.50 ± 2.00*	*8.52 ± 3.13*	*<0.001*
**New born monitoring**	**8.25 ± 2.22**	**8.13 ± 1.66**	**0.708^{NS}**
Handwashing with soap	9.29 ± 2.47	8.74 ± 3.10	0.037
Danger signs assessment in the mother and the child	9.62 ± 1.60	9.22 ± 2.11	0.021
Chlorhexidine gel application	5.47 ± 4.89	4.10 ± 4.61	0.101^{NS}
Advice on exclusive breastfeeding	9.34 ± 2.30	9.36 ± 2.19	0.910^{NS}
Advice on complementary feeding	7.64 ± 3.99	8.21 ± 3.16	0.062^{NS}
Advice on the use of long-lasting insecticide-treated nets	8.69 ± 3.27	8.55 ± 3.16	0.621^{NS}

Advice on feeding of the breastfeeding mother	7.30 ± 4.26	7.78 ± 3.56	0.170[NS]
Family planning counselling	8.36 ± 3.50	8.34 ± 3.26	0.945[NS]
Advice on postnatal consultation	8.03 ± 3.86	7.49 ± 3.76	0.109[NS]
Advice on food, personal and environmental hygiene	7.63 ± 3.92	7.85 ± 3.31	0.473[NS]
Advice on vaccination	8.18 ± 3.69	8.60 ± 3.14	0.151[NS]
Advice on maintaining the temperature of the newborn	8.52 ± 3.29	8.48 ± 3.14	0.897[NS]
**Family planning**	8.78 ± 1.96	8.28 ± 2.15	**0.009**
Handwashing with soap	8.30 ± 3.67	7.89 ± 3.92	0.234[NS]
Compliance with the GATHER milestones	9.90 ± 2.13	8.27 ± 2.95	0.001
Use of the eligibility checklist	8.81 ± 3.10	8.21 ± 3.46	0.046
Respect for confidentiality	8.91 ± 2.96	8.23 ± 3.43	0.020
**IYCF promotion**	**6.20 ± 3.60**	**7.18 ± 3.02**	**0.003**
Compliance with the steps of the IYCF evaluation	6.83 ± 3.80	7.54 ± 3.24	0.020
Compliance with the steps in facilitating the support group	6.07 ± 4.45	7.11 ± 3.63	0.007
Compliance with the steps of the nutritional demonstration	5.17 ± 4.71	6.73 ± 3.95	<0.001
**Hygiene and sanitation promotion**	**8.67 ± 1.21**	**7.67 ± 1.81**	**<0.001**
Mastery of handwashing technique	9.68 ± 1.39	8.94 ± 2.38	<0.001
Knowledge of critical handwashing moments	9.64 ± 1.38	9.35 ± 1.76	0.035
Water treatment by aquatabs	9.73 ± 1.28	9.28 ± 2.10	0.005
Water treatment with bleach	8.73 ± 2.86	7.50 ± 3.18	< 0.001
Water treatment by filter	9.44 ± 1.85	7.72 ± 3.29	< 0.001
Water treatment by settling	8.85 ± 2.79	6.12 ± 4.02	< 0.001
Mastery of well treatment technique	4.21 ± 3.85	4.82 ± 3.93	0.097[NS]

GATHER: Greet, Ask, Tell, Help, Explain and Return; iCCM: integrated community case management; IYCF: Infant and Young Child Feeding; NS: Not-significant result; MUAC: middle upper arm circumference; RDT: rapid diagnostic test for malaria; SD: standard deviation.

Table B-2 - Average score obtained by the community health workers on the nutrition-specific supervisions in Kita district from January to October 2018.

**KITA**<br>(High supervision)	**Average score**<br>Mean ± SD
**Equipment availability**	**7.50 ± 3.30**
Anthropometric equipment	*6.80 ± 4.67*
Medical equipment	*8.21 ± 3.83*
**Other materials**	**4.01 ± 0.91**
Register sheets	*9.73 ± 1.63*
Patient cards, growth charts, reference sheets, etc.	*8.17 ± 3.87*
Protocols	*3.70 ± 4.83*
Educational material and counseling cards	*9.13 ± 2.82*
Communication material	*8.71 ± 3.36*
**Pharmacy stock**	**2.68 ± 1.32**
Adequate, clean and ventilated stock/pharmacy conditions	*6.82* ± 4.66
Stock cards for nutritional and routine treatment drugs inputs	*6.40* ± 4.85
Sufficient quantity of nutritional and routine drugs inputs and iodine testers in stock and no stock-out	*6.31* ± 4.87
Inventory carried out monthly	*6.98* ± 4.60
**Safe drinking water available**	**6.43 ± 4.80**
**Identification of danger signs**	**9.92 ± 0.64**
Checking if child is <2 months	*9.66 ± 1.81*
Checking not gain weight (2-6 months)	*9.87 ± 1.15*
Checking severe weight loss (6-12 months)	*9.91 ± 0.94*
Unable to drink or suckle assessment	*9.87 ± 1.15*
Vomiting assessment	*9.87 ± 1.15*
Seizure or convulsing assessment	*9.91 ± 0.94*
Unconscious or unresponsive assessment	*9.82 ± 1.32*
Oedema assessment	*9.89 ± 1.05*
Severe palmar pallor assessment	*9.91 ± 0.94*
Difficult breathing assessment	*9.93 ± 0.81*

Spontaneous bleeding assessment	*9.91 ± 0.94*
Urine scarce and color assessment	*9.84 ± 1.24*
Check if unable to sit or stand upright	*9.84 ± 1.24*
Check if gets sicker despite home care	*9.87 ± 1.15*
Check if illnesses lasting 14 days or more	*9.89 ± 1.05*
Dehydration assessment	*9.84 ± 1.24*
Red MUAC assessment	*9.88 ± 1.09*
Red MUAC and appetite test failure	*9.86 ± 1.18*
Blood in the stool assessment	*9.87 ± 1.15*
Stools too liquid assessment	*9.80 ± 1.40*
**Systematic screening of other diseases**	**9.73 ± 1.62**
**Admission & discharge criteria application**	**8.78 ± 2.63**
Admission criteria application	*9.07 ± 0.29*
Discharge criteria application	*7.40 ± 4.39*
**Appetite test performance**	**8.65 ± 2.88**
Correct quantity of RUTF given	*8.56 ± 3.51*
Monitoring the mother and result noted	*8.72 ± 3.34*
**Nutritional treatment**	**8.96 ± 3.06**
**Systematic medical treatment**	**7.42 ± 4.38**
**IYCF promotion**	**4.92 ± 3.38**
Promotion and advises if child <2months	*4.79 ± 5.00*
Counselling sessions pregnant women	*6.92 ± 4.62*
Nutritional demonstration	*2.63 ± 4.41*

Supplementary C

Table C-1-Univariate linear regression analysis to assess the influence of the number of supervisions received by the community health workers and their quality of performance on the proportion of children discharge as cured.

**iCCM supervision (Kita and Kayes districts)**	**N**	**B [C.I. 95%]***	**p value**
Number of supervisions received	100	0.114 [-1.931 – 2.158]	0.912
Clinical examination	93	0.536 [-6.179 – 7.252]	0.874
New born monitoring	61	-1.556 [-4.462 – 1.350]	0.288
Family planning	96	-1.703 [-4.231 – 0.825]	0.184
IYCF promotion	85	-1.288 [-2.990 – 0.414]	0.136
Hygiene and sanitation promotion	88	-2.566 [-6.212 – 1.081]	0.165
**Nutrition-specific supervision (Kita district)**	**N**	**B [C.I. 95%]***	**p value**
Number of supervisions received	61	0.649 [-2.517 – 3.816]	0.683
Identification of danger signs	61	0.020 [-18.076 – 18.117]	0.998
Systematic screening	61	0.032 [-8.229 – 8.292]	0.994
Admission and discharge criteria application	61	-1.298 [-4.233 – 1.637]	0.380
Nutritional treatment	61	1.490 [-1.723 – 4.703]	0.357
Systematic medical treatment	61	0.065 [-1.770 – 1.900]	0.944

C.I.: confidence interval; CHWs: community health workers; iCCM: integrated community case management; IYCF: infant and young child feeding; N: number of CHWs included in the analysis (those that have data for both supervision variables and treatment performance).

*Dependent variable: Percentage of children discharge as cured from the total treated during study period.

Table C-2-Univariate logistic regression analysis to assess the influence of the number of supervisions received by the community health workers and their quality of performance on the probability of reaching 75% of cured children which is the minimum stablished by the SPHERE Standards in disaster response (https://spherestandards.org/humanitarian-standards/)

**iCCM supervision (Kita and Kayes districts)**	**N**	**β [C.I. 95%]***	**p value**
Number of supervisions received	100	1.314 [0.670 – 2.578]	0.427
Clinical examination	93	1.432 [0.856 – 2.396]	0.171
New born monitoring	61	0.961 [0.695 – 1.329]	0.961
Family planning	96	0.920 [0.697– 1.216]	0.559
IYCF promotion	85	0.905 [0.757 – 1.082]	0.273
Hygiene and sanitation promotion	88	1.050 [0.718 – 1.537]	0.801
**Nutrition-specific supervision (Kita district)**	**N**	**β [C.I. 95%]***	**p value**
Number of supervisions received	61	1.057 [0.781 – 1.431]	0.720
Identification of danger signs	61	0.704 [0.106 – 4.677]	0.716
Systematic screening	61	1.195 [0.555 – 2.576]	0.649
Admission and discharge criteria application	61	0.816 [0.571 – 1.166]	0.264
Nutritional treatment	61	1.130 [0.830 – 1.537]	0.437
Systematic medical treatment	61	0.954 [0.796 – 1.142]	0.605

C.I.: Confidence interval; CHWs: Community Health Workers; iCCM: Integrated Community Case Management; IYCF: Infant and young child feeding; N: Number of CHWs included in the analysis (those that have data for both supervision variables and treatment performance).

*Dependent variable: Percentage of children discharge as cured from the total treated during study period.
